# Workload associated with mrsa control in surgery: a prospective study alongside a controlled clinical trial

**DOI:** 10.1186/2047-2994-4-S1-P188

**Published:** 2015-06-16

**Authors:** S Bahrami, AS Lee, S Harbarth, S Malhotra-Kumar, C Brun-Buisson, I Durand-Zaleski, MOSAR WP9 and WP4 working groups

**Affiliations:** 1 AP-HP, Paris, France; 2 Royal Prince Alfred Hospital, Sydney, Australia; 3 University of Geneva Hospitals, Switzerland; 4 University of Antwerp, Belgium; 5 Inserm U657, Paris, France

## Introduction

Controversies regarding control of endemic methicillin-resistant *Staphylococcus aureus* (MRSA) stem in part from the paucity of data available on the actual costs of implementing MRSA control strategies.

## Objectives

To estimate the workloads associated with screening and hand hygiene (HH) promotion interventions for MRSA control.

## Methods

Prospective evaluation of costs associated with MRSA control interventions, alongside the MOSAR–Surgery multicenter intervention trial [1], emphasizing the workloads associated with interventions. Interventions under study were (1) universal MRSA screening with contact precautions and decolonization (4 hospitals) and (2) enhanced HH promotion (4 hospitals); 2 hospitals implemented a combined strategy using targeted MRSA screening.

Workloads were estimated from a hospital perspective, using a top-down approach, distinguishing infection control (IC) and ward staff duties, set-up and routine activities, and excluding research-driven tasks.

## Results

In the screening arm (13 wards; mean, 27.8 ± 10.4 beds), set-up required 2.6 ± 0.58 weeks of work from the IC team, and the mean annual workload was 20.5 weeks or 8.62 ± 4.39 weeks for a 10-bed ward. In the HH promotion arm (13 wards, 87.8 ± 79.5 beds), set-up required 2.77 ± 1.13 weeks, and mean annual workload was 12.1 weeks (3.29 ± 3.72 per 10 beds). In the combined arm (7 wards, 44.9 ± 24.6 beds), set-up required 7.0 ± 1.93 weeks, and mean annual workload was 23.7 weeks (5.94 ± 1.84 weeks per 10 beds). The burden on ward staff was relatively limited in most wards.

## Conclusion

Workload associated with the MRSA screening strategy is relatively homogenous and predictable. Investment in the HH promotion strategy showed large variations between centers and was maximal for the successful combined intervention. Further research is needed on the optimal implementation of HH promotion interventions.

## Disclosure of interest

S. Bahrami: None declared, A. Lee: None declared, S. Harbarth Grant/Research support from: Geneva University Hospitals, B. Braun, Pfizer, Speaker’s bureau of: BioMerieux, Pfizer, Conflict with: Scientific Advisory Board, Destiny Pharma, DaVolterra, BioMérieux, S. Malhotra-Kumar: None declared, C. Brun-Buisson: None declared, I. Durand-Zaleski: None declared.

## References

[B1] Lee AS Cooper BS Malhotra-Kumar S BMJ Open 2013 3 e003126 24056477 10.1136/bmjopen-2013-003126 PMC3780302

